# Effect of divalent ions on cariogenic biofilm formation

**DOI:** 10.1186/s12866-020-01973-7

**Published:** 2020-09-16

**Authors:** Elena Laura Steiger, Julia Rahel Muelli, Olivier Braissant, Tuomas Waltimo, Monika Astasov-Frauenhoffer

**Affiliations:** 1 Department for Oral Health & Medicine, University Center for Dental Medicine (UZB), University of Basel, Mattenstrasse 40, 4058 Basel, Switzerland; 2 Center of Biomechanics and Biocalorimetry, c/o Department of Biomedical Engineering (DBE), University of Basel, Gewerbestrasse 14, 4123 Allschwil, Switzerland; 3 Department Research, University Center for Dental Medicine (UZB), University of Basel, Mattenstrasse 40, 4058 Basel, Switzerland

**Keywords:** Caries, Biofilm, Mutans streptococci, Divalent ions

## Abstract

**Background:**

Divalent cations are able to interact with exopolysaccharides (EPS) and thus are capable to modify the structure and composition of dental biofilm. At the moment, little is known about the adsorption of metals by cariogenic EPS; thus, the aim of the present study was to evaluate the effect of divalent ions (calcium, magnesium, and zinc) on the growth and biofilm formation of mutans streptococci and on the dissolution of hydroxyapatite as well as to investigate their binding to the bacterial EPS.

**Results:**

*S. mutans* strains used in this study show the highest tolerance towards calcium of the ions tested. Growth parameters showed no differences to control condition for both strains up to 100 mM; revealing natural tolerance to higher concentration of calcium in the surroundings. Although excessive levels of calcium did not impair the growth parameters, it also did not have a positive effect on biofilm formation or its binding affinity to EPS. Magnesium-saturated environment proved to be counterproductive as strains were able to dissolve more Ca^2+^ from the tooth surface in the presence of magnesium, therefore releasing excessive amounts of Ca^2+^ in the environment and leading to the progression of the disease. Thus, this supports the idea of self-regulation, when more Ca^2+^ is released, more calcium is bound to the biofilm strengthening its structure and however, also less is left for remineralization. Zinc inhibited bacterial adhesion already at low concentrations and had a strong antibacterial effect on the strains as well as on calcium dissolution; leading to less biofilm and less EPS. Additionally, Zn^2+^ had almost always the lowest affinity to all EPS; thus, the unbound zinc could also still remain in the surrounding environment and keep its antimicrobial properties.

**Conclusion:**

It is important to maintain a stable relationship between calcium, magnesium and zinc as excessive concentrations of one can easily destroy the balance between the three in cariogenic environment and lead to progression of the disease.

## Background

Dental caries is one of the most frequent infectious multifactorial diseases worldwide, characterized as a progressive dissolution and demineralization of tooth structure; when left untreated it can lead to an impaired quality of life [1–3]. The development depends on various aspects e.g. personal and social-economic factors (e.g. education, knowledge, behavior, income), genetic and environmental factors (e.g. pH-value, buffer capacity, composition and flowrate of saliva) [4]. Furthermore, studies have shown that frequent intake of high sugar content food and beverages increases the personal caries risk [5–7]. In such conditions, acidogenic bacteria, like mutans streptococci, are capable of generating an acidic shift in the dental biofilm which results in the demineralization of the hard tissues of the teeth [1–3, 8, 9]. This manifestation occurs only if there is an imbalance between the protective and the pathogenic factors inside the oral biofilm. Nevertheless, the progression can only be arrested by preventing further biofilm formation [10]. Sucrose is the most commonly consumed carbohydrate and has proven to be very cariogenic [9, 11–13], especially in combination with a microflora that contains high numbers of *Streptococcus mutans* [14, 15]. Moreover, also monosaccharides, like glucose, fructose, galactose, or disaccharides, like lactose and maltose, play a significant role in the development of caries [7, 13].

In most biofilms, and hence in dental plaque, the microbes make up only for about 10% of the dry mass while rest is extracellular matrix [16]. Decisive parts of the matrix in the dental biofilm are the extracellular polysaccharides (EPS), which in caries lesions are predominantly produced by *S. mutans* [17]. The development of EPS depends on various factors such as pH, temperature, time, and the composition of the medium, in which the sources of carbon, nitrogen, and divalent ions play an important role [18]. Since EPS contains negative charged functional groups and has the potential in removing heavy metal ions from solutions, there is the possibility that divalent ions can bind EPS as well and therefore change its whole structure and function [19].

The growth of the biofilm, and thus the aggregation of EPS, depends on specific environmental signals [20]. The study of Cheng et al. showed that SnF_2_-containing toothpastes reduce the amount of EPS in the dental plaque and consequently change the entire structure of the cariogenic biofilm s due to a change in the metabolism of *S. mutans* after the uptake of tin [21]. Since EPS is produced by streptococcal glucosyltransferases, it can be assumed that its development can be reduced and thus, the growth of biofilm stopped [22]. Therefore, it is presumed that the development or the progression of dental caries can be modified by the application of certain cations. Among those ions, it is expected that Ca, Mg and Zn might have an important role as they are naturally present in the upper surfaces of teeth.

Calcium is capable to affect the biofilm production through diverse mechanisms [20]. High concentrations of Ca^2+^ have shown to enhance biofilm formation in a dental lesion [8]. The calcium in teeth can act as a mineral buffer; however, when released, it reduces the mineral dissolution [23]. Consequently, it can be concluded that the demineralization can be limited, and also that the dental biofilm plays a considerable role in the calcium regulation [8, 24].

Similarly, magnesium levels in teeth tend to decrease with age, leading to a change in the mineralized tissues and thus a reduction in its physical properties such as hardness [25]. In order to prevent this, dentifrices containing magnesium have been developed to protect the teeth from external acids, ultimately resulting in an increased hardness of the whole tooth framework [25, 26].

Finally, zinc is also a cation that can lead to changes in the development and progression of dental lesions. It is naturally found as a trace element in saliva (mean concentration 3.95 mg/L, [27]), dental biofilm and also in the teeth [28, 29]. Zn^2+^ is able to support the control of biofilm development, reduce malodour and also inhibit calculus formation [29]. Thus, zinc oxide and zinc citrate are often present in toothpastes or in mouth rinses as they inhibit biofilm formation by reducing glucan synthesis; however, that cannot prevent the formation of tooth decays on its own [30].

Divalent cations are able to interact with EPS and thus are capable to modify the structure and composition of dental biofilm. At the moment, little is known about the adsorption of metals by cariogenic EPS, as it depends on numerous factors. In addition, the analysis of the multiple interactions between EPS and the divalent ions is difficult because of the low availability of the predictive tools [31].

Thus, the aim of the present study was to evaluate the effect of divalent ions (calcium, magnesium, and zinc) on the growth and biofilm formation of mutans streptococci and on the dissolution of hydroxyapatite as well as to investigate their binding to the bacterial EPS.

## Results

### Effect of divalent ions on the growth of mutans streptococci

The effect of divalent ions on the growth of *S. mutans* was investigated by using three different dietary sugars (glucose, sucrose and fructose) and three different types of divalent ions (Ca^2+^, Mg^2+^ and Zn^2+^) in combination with chloride (CaCl_2_, MgCl_2_, and ZnCl_2_).

**Fig. 1:**
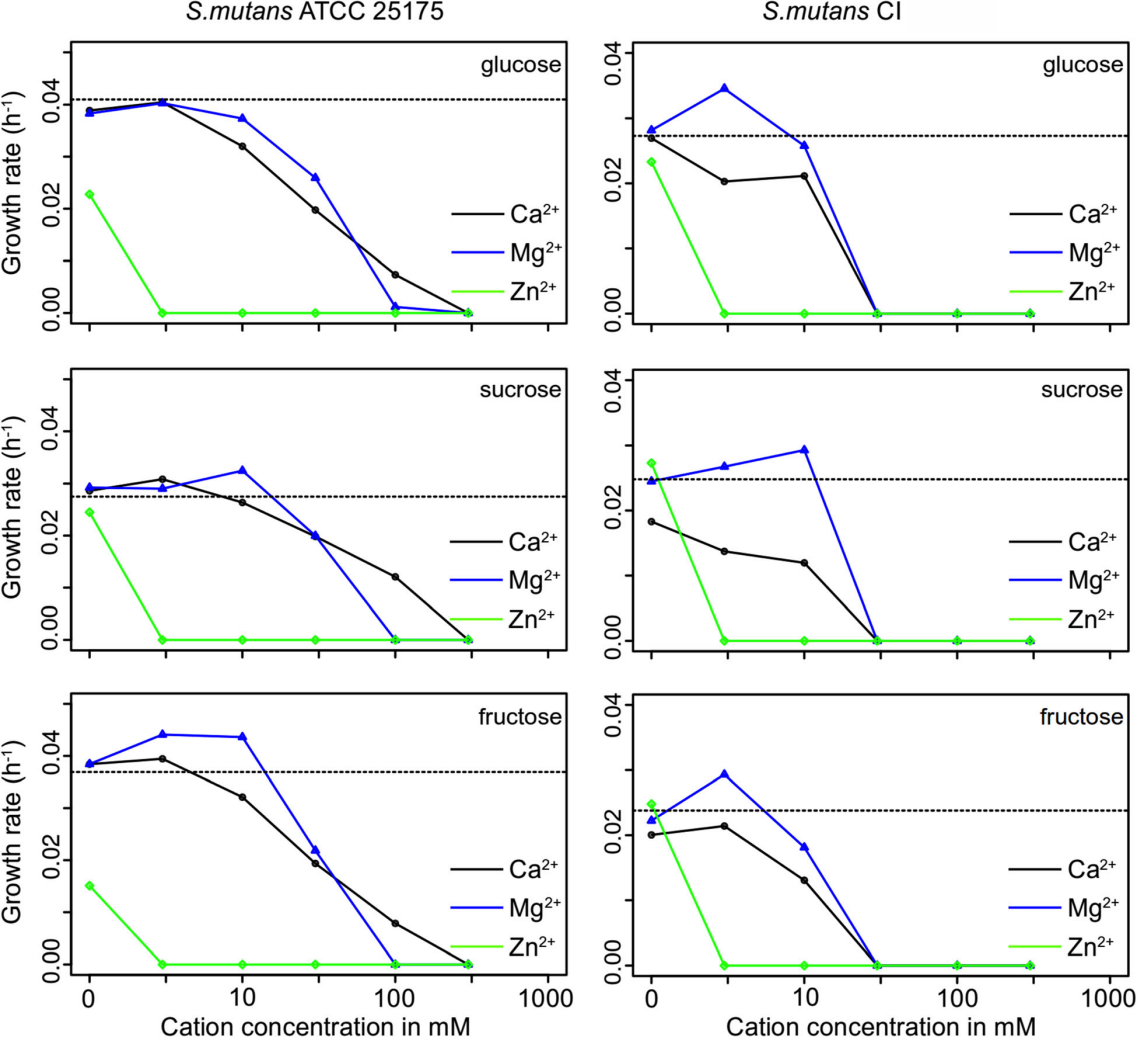
Growth rate of the *S. mutans* strains when incubated with divalent ions in the presence of different carbohydrate sources

Growth rate (Fig. 1) was significantly affected for the type strain of *S. mutans* at 30 mM concentration of CaCl_2_ when supplemented carbohydrate was glucose, with sucrose the growth rate was affected in the presence of CaCl_2_ higher than 30 mM while MgCl_2_ and ZnCl_2_ resulted in no changes in growth rate parameter. When fructose was used as a supplementing carbohydrate, the growth rate was affected by CaCl_2_ higher than 30 mM and ZnCl_2_ over 1 mM; however, MgCl_2_ showed no effect on this parameter. Interestingly, the growth rate of *S. mutans* clinical isolate (CI) was affected only when glucose and CaCl_2_ were present; all other combinations showed no significant decrease in this parameter.

The results for growth parameter lag time are shown in Table 1 for both strains. MgCl_2_ and ZnCl_2_ were able to prolong the lag time significantly at 30 mM and 1 mM concentration, respectively; while CaCl_2_ showed only minimal effect at 100 mM with glucose and no effect at all with the other carbohydrates in the samples where growth was detected over 48 h. *S. mutans* CI showed no difference in lag times in all three carbohydrate solutions when incubated with CaCl_2_, while significant prolongation of the parameter was observed at 10 mM with MgCl_2_ and at 1 mM with ZnCl_2_.

**Table 1:** Lag time of the *S. mutans* strains when incubated with divalent ions in the presence of different carbohydrate sources

Carbohydrate	Added agent	lag time (h)						
		0 mM	1 mM	3 mM	10 mM	30 mM	100 mM	200 mM
***S. mutans ATCC 25175***								
Glucose	CaCl_2_	9.58 ± 0.14	9.69 ± 0.20	9.77 ± 0.14	9.98 ± 0.09	9.70 ± 0.47	11.65 ± 0.63	ND
Sucrose		7.91 ± 0.79	7.79 ± 1.14	7.94 ± 0.81	8.11 ± 0.45	9.26 ± 0.88	9.77 ± 0.27	ND
Fructose		6.90 ± 0.56	7.61 ± 0.53	7.50 ± 0.49	7.30 ± 0.49	8.67 ± 0.20	8.39 ± 0.24	ND
Glucose	MgCl_2_	9.58 ± 0.14	9.68 ± 0.19	9.56 ± 0.04	10.30 ± 0.49	21.92 ± 0.97	24.11 ± 0.00	ND
Sucrose		7.91 ± 0.79	7.94 ± 0.96	8.09 ± 0.92	8.91 ± 1.00	23.71 ± 2.62	ND	ND
Fructose		6.90 ± 0.56	7.28 ± 0.64	7.56 ± 0.59	8.56 ± 0.35	16.40 ± 0.95	ND	ND
Glucose	ZnCl_2_	9.58 ± 0.14	17.82 ± 2.46	ND	ND	ND	ND	ND
Sucrose		7.91 ± 0.79	13.86 ± 1.17	ND	ND	ND	ND	ND
Fructose		6.90 ± 0.56	17.28 ± 2.01	ND	ND	ND	ND	ND
***S. mutans CI***								
Glucose	CaCl_2_	12.10 ± 0.44	11.76 ± 0.96	12.84 ± 0.56	11.83 ± 0.46	ND	ND	ND
Sucrose		10.11 ± 0.73	10.83 ± 0.71	10.52 ± 0.85	10.89 ± 0.44	ND	ND	ND
Fructose		12.83 ± 1.37	13.02 ± 1.82	12.61 ± 0.70	13.47 ± 1.26	ND	ND	ND
Glucose	MgCl_2_	12.10 ± 0.44	11.86 ± 0.60	12.14 ± 0.50	16.96 ± 1.66	ND	ND	ND
Sucrose		10.11 ± 0.73	10.27 ± 0.57	9.98 ± 0.62	15.75 ± 1.25	ND	ND	ND
Fructose		12.83 ± 1.37	12.81 ± 1.77	12.94 ± 1.33	21.38 ± 3.94	ND	ND	ND
Glucose	ZnCl_2_	12.10 ± 0.44	15.76 ± 0.92	ND	ND	ND	ND	ND
Sucrose		10.11 ± 0.73	17.91 ± 0.64	ND	ND	ND	ND	ND
Fructose		12.83 ± 1.37	16.36 ± 1.80	ND	ND	ND	ND	ND

### Cariogenic dissolution of hydroxyapatite

**Fig. 2:**
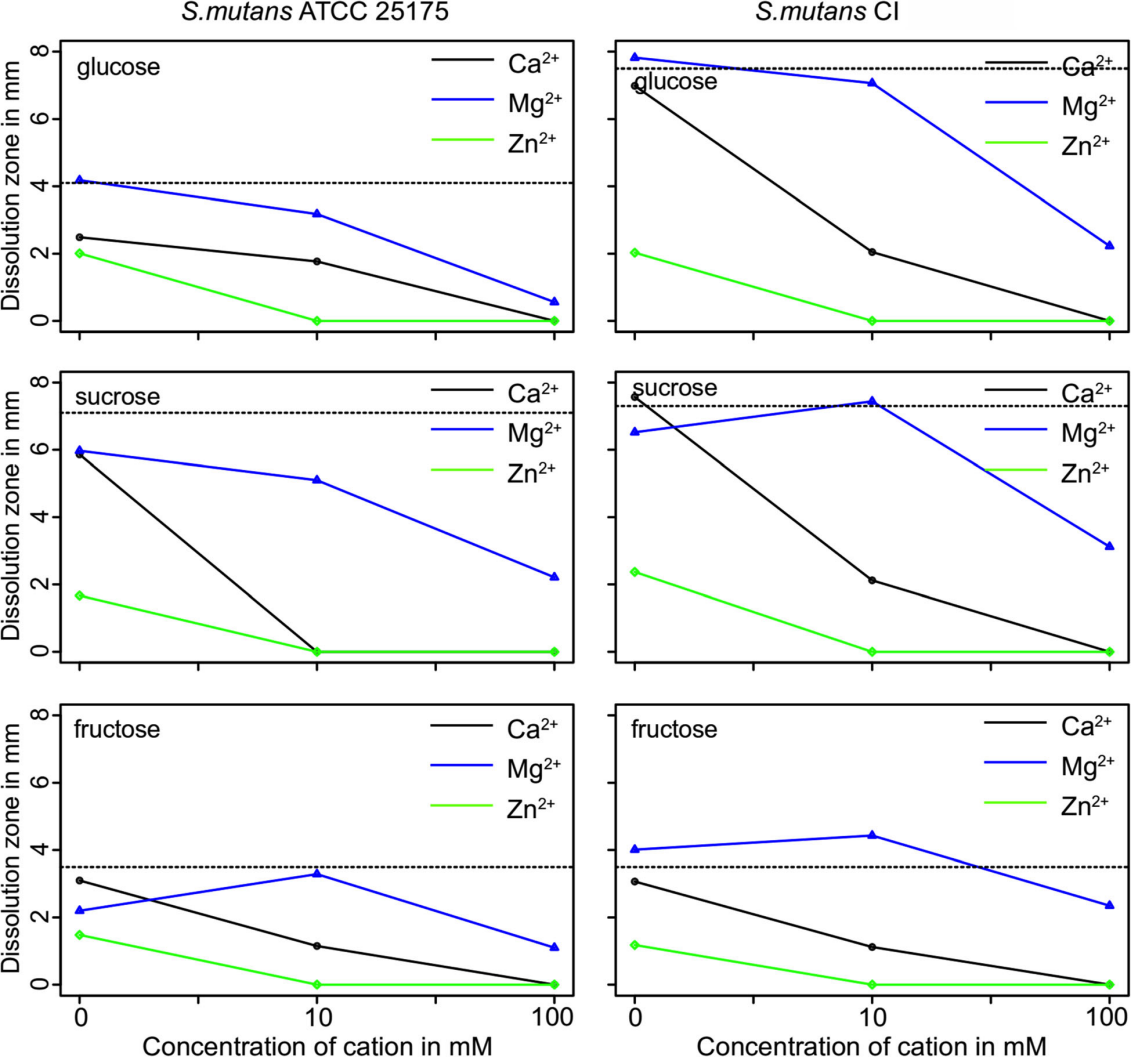
Dissolution of hydroxyapatite over 10 d by *S. mutans* strains. (mm; mean ± SD)

Dissolution of hydroxyapatite was measured in the presence of divalent ions (Fig. 2). There is a dose-dependency between the size of the dissolution zone and the concentration of calcium and zinc for all carbohydrates used in this study. Magnesium seems to be promoting the dissolution of hydroxyapatite for both species. However, a significant reduction in hydroxyapatite is first achieved at 100 mM magnesium.

### Microscopic analysis of formed biofilm

**Fig. 3:**
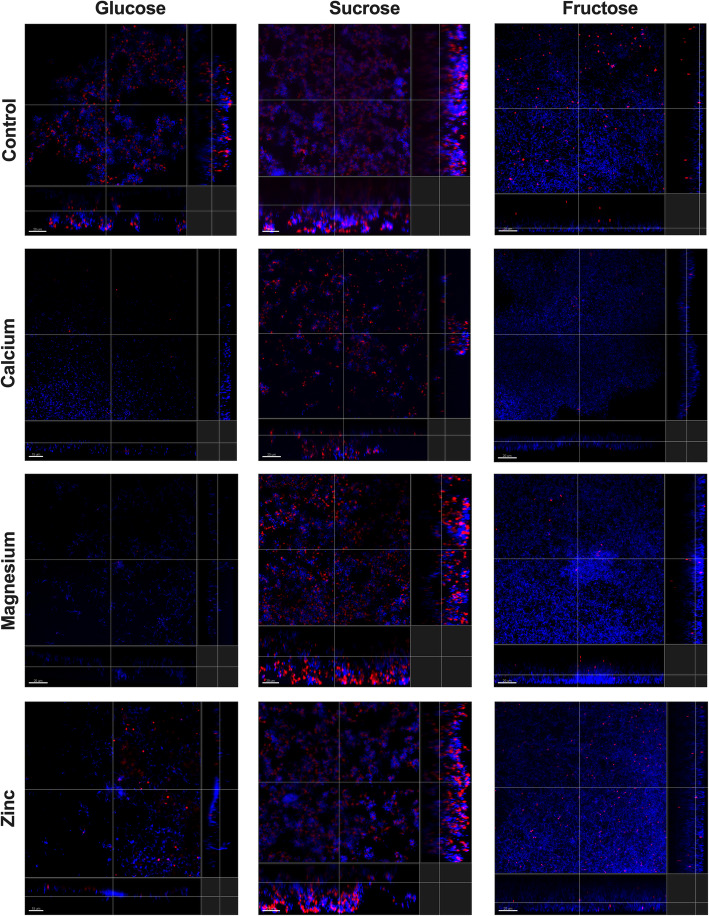
Formation of *S. mutans* ATCC 25175 biofilms on hydroxyapatite disks in the presence of divalent ions

**Fig. 4:**
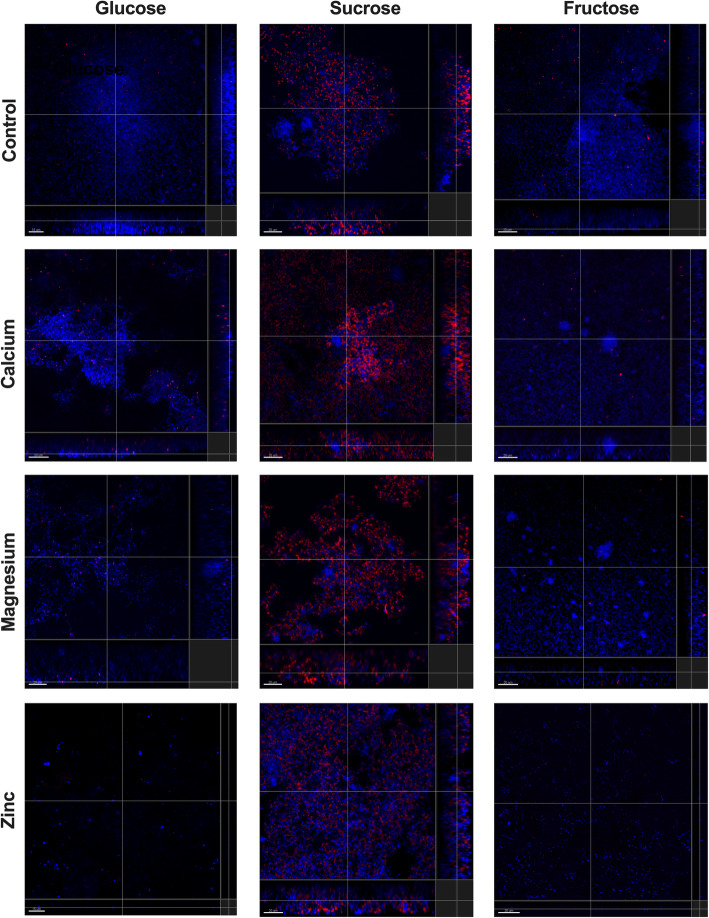
Formation of *S. mutans* CI biofilms on hydroxyapatite disks in the presence of divalent ions

The highest concentrations of divalent ions allowing still bacterial survival (and growth based on Fig. 1 and Table 1), were selected to check the effect of biofilm formation on hydroxyapatite (HA) disks (Fig. 3, Fig. 4). EPS was visible in all samples that had sucrose as supplemented carbohydrate. Interestingly, while Ca^2+^ seems to promote the EPS formation by *S. mutans CI*, in *S. mutans* type strain the least EPS was detected in the presence of this divalent ion. With fructose both strains form a very thin layer of adhered cells and no EPS could be observed. Adding glucose to the solution seems to have no effect in promoting growth, in case of *S. mutans* CI almost no adherent cells were detected in the presence of Zn^2+^, while adhesion seems to be more inhibited by Ca^2+^ and Mg^2+^ for *S. mutans* type strain.

### Analysis of the binding of the divalent ions to EPS

Yield of EPS was determined by sulphuric acid assay and the results for glucose, sucrose, and fructose in case of *S. mutans* type strain were 0.74 mg/mL, 1.22 mg/mL, and 0.70 mg/mL, respectively. For *S. mutans* CI, similar results were measured: 0.73 mg/mL, 1.61 mg/mL, and 0.83 mg/mL respectively.

The binding affinity of EPS to the tested divalent ions seems to be strain-related for calcium and magnesium, while the affinity to zinc is comparable between those two strains (Table 2). The EPS of *S. mutans* CI has a higher binding affinity towards calcium and magnesium than the type strain and the results are in the same order of magnitude than the ions’ affinity to dextran. Other parameters detected by isothermal titration calorimetry (ITC) show comparable results between the strains. However, one has to keep in mind that such result can only be an estimate as the complexity of the many binding sites of EPS is not reflected by the single step equilibrium model.

**Table 2:** Parameters assessed by isothermal titration calorimetry (ITC) for EPS extracted from *S. mutans* strains using different carbohydrate source

Carbohydrate	Titration agent	***S. mutans ATCC 25175***			
		k	ΔH_1_ (kJ/mol)	ΔG_1_ (kJ/mol)	ΔS_1_ (J/(k x mol))
Glucose	CaCl_2_	3.7x10^3^ (±5.0x10^2^)	−58.5 (±3.6)	−20.3	−127.9
Sucrose		3.1x10^3^ (±7.9x10^2^)	−58 (±14)	−19.9	− 126.4
Fructose		9.2x10^3^ (±1.7x10^3^)	−25.6 (±1.2)	−22.6	−9.9
Glucose	MgCl_2_	7.9x10^3^ (±1.5x10^3^)	−43.6 (±2.6)	−22.2	−71.8
Sucrose		5.3x10^3^ (±2.3x10^3^)	7.8 (±1.4)	−21.5	97.1
Fructose		1.9x10^3^ (±1.3x10^3^)	22 (±40)	−19.0	137.6
Glucose	ZnCl_2_	1.4x10^4^ (±0.72x10^4^)	16 (± 2.8)	−23.9	133.2
Sucrose		3.9x10^3^ (±0.71x10^3^)	17 (±12)	−20.7	220.9
Fructose		3.6x10^3^ (±0.66x10^3^)	31 (±5.6)	−20.5	172.1
Carbohydrate	Titration agent	***S. mutans CI***			
		k	ΔH_1_ (kJ/mol)	ΔG_1_ (kJ/mol)	ΔS_1_ (J/(k x mol))
Glucose	CaCl_2_	1.1x10^4^ (±3.2x10^3^)	−29.0 (±2.0)	−23.2	−19.6
Sucrose		4.9x10^4^ (±2.0x10^3^)	4.78 (±0.20)	−26.8	105.9
Fructose		1.2x10^4^ (±2.2x10^3^)	−6.0 (±0.25)	−24.2	58.6
Glucose	MgCl_2_	1.4x10^4^ (±0.44x10^4^)	9.5 (±0.73)	−23.9	110.9
Sucrose		1.5x10^4^ (±0.91x10^4^)	4.4 (±0.65)	−24.1	94.9
Fructose		1.7x10^4^ (±1.4x10^4^)	6.2 (±1.5)	−24.4	101.7
Glucose	ZnCl_2_	4.8x10^3^ (±0.79x10^3^)	42 (±6.3)	−21.2	209.2
Sucrose		4.2x10^3^ (±1.1x10^3^)	34 (±4.8)	−20.9	182.8
Fructose		4.8x10^3^ (±0.79x10^3^)	42 (±6.3)	−21.2	209.2
	Titration agent	***Dextran***			
		k	ΔH_1_ (kJ/mol)	ΔG_1_ (kJ/mol)	ΔS_1_ (J/(k x mol))
	CaCl_2_	2.6x10^4^ (±7.3x10^3^)	5.60 (±0.21)	−25.2	102.2
	MgCl_2_	3.2x10^4^ (±8.6x10^3^)	8.29 (±0.28)	−25.9	113.7
	ZnCl_2_	2.1x10^4^ (±5.2x10^3^)	5.99 (±0.25)	−24.7	102.9

Additional experiments were conducted where EPS was saturated by magnesium and thereafter the titration was conducted with calcium. No calcium could be bound for both strains as magnesium had saturated all the binding sites.

## Discussion

The interactions between the hard tissues of the teeth and the environment of the oral cavity depend on salivary proteins and enzymes among others but also on dissolved ions [32, 33]. Calcium dissolved in saliva is known to influence the process of de- and remineralization of the tooth enamel which in combination with cariogenic microbes can lead to caries and to a reduction of the general health condition [34, 35]. Therefore, it is important to understand the interactions between microorganisms, components of saliva, and the tooth mineral surface, to ensure a good oral health. Thus, this study investigates the effect of divalent cations on the formation of cariogenic biofilm in order to find the importance of these ions and how to prevent EPS and biofilms formation in different conditions. In order to investigate the effect of virulence we used a type strain but also a clinical isolate, that has been studied earlier as the structure and composition of EPS is affected by the characteristics acquired by the strain to survive in certain environments. Thus, here a type strain was used to provide as much reproducibility as possible as the strain is well-characterized and in comparison, a clinical isolate that has mutated already to survive in highly cariogenic lesions with low pH [8].

The exoenzymes of *S. mutans* are capable of forming EPS in attendance of glucose, fructose, and sucrose, a disaccharide of glucose and fructose [8, 36]. As the main components of the matrix are EPS, they are found to be a substantial virulence factor associated with dental caries with some variances in their functional groups due to differences in the carbohydrates that were fermented [36]. However, previous studies have mostly used EPS formed in the presence of sucrose; thus, here also the aspect how carbohydrate influence the EPS abundance to divalent ions was assessed. Due to the primary binding sites formed by EPS, various constituents of saliva are able to affect the microbial adhesion to the dental plaque by influencing the electrostatic interactions [8, 37]. Therefore, the selective adhesion and accumulation of pathogenic streptococci, but also the binding of biologically important cations such as Ca^2+^, Mg^2+^, Fe^2+/3+^ and Zn^2+^, can take place. EPS have numerous negatively charged functional groups (carboxyl, phosphate and amine groups) [8, 38] that are capable to interact with positive charged cations, thereby also with the three divalent ions used in this study (Mg^2+^, Zn^2+^ and Ca^2+^) [19, 39]. Thus, if several of the negative functional groups were bound by these cations, the structure and function of the entire biofilm could be changed and consequently reduce (or in worst case possibly enhance) the development of caries.

Calcium is predominantly known as an important regulatory ion for almost all of the pro- and eukaryotic cells [40]. Along with other ions, calcium plays a specific role in the cariogenic biofilm formation of caries-related species. Demineralization of tooth surface always results in a calcium release and an increase of the calcium concentration in the biofilm and its close surroundings, which might have a negative effect on the microbes [8, 41]. *S. mutans* strains used in this study show the highest tolerance towards calcium of the three ions tested. Both of the growth parameters lag time (Table 1) and growth rate (Fig. 1) of the strains showed no differences to control condition up to 100 mM concentration; meaning the cariogenic strains have a natural tolerance to higher concentration of calcium in the surroundings. Although excessive levels of calcium did not impair the growth parameters, it also did not have a positive effect on biofilm formation (Fig. 3, Fig. 4) or its binding affinity to EPS (Table 2). While in many aspects’ calcium revealed similar action to magnesium in this study (Table 2), it is less prone to promote the growth of biofilm (Fig. 3, Fig. 4) and the cariogenic effects of mutans steptococci (Fig. 2) as magnesium seems to be doing.

Magnesium is capable of altering the cell structure, thereby preventing bacterial adhesion, which results in a disruption of the development of biofilm [42]. Interestingly, here no such effect was seen. Magnesium promoted dissolution of calcium from hydroxyapatite (Fig. 2) as well as the formation of biofilms (Fig. 3, Fig. 4). That is interesting as no such effect is seen in vivo. That; however, might be contributed to the fact that the average Mg^2+^ concentration in dental plaque is lower than that of Ca^2+^. Moreover, Mg^2+^ acts as a competitive inhibitor and can reduce through ionic interactions Ca^2+^ binding by approximately 15% [43]. In this study it could be shown that Mg^2+^-saturated EPS could not bind any longer any Ca^2+^ ions; this might be due to the small differences between magnesium and calcium binding affinity suggesting that magnesium could not replace the calcium at least at low concentrations. Consequently, a production of a Mg^2+^-saturated environment can be counterproductive as mutans streptococci are able to dissolve more Ca^2+^ from the tooth surface in the presence of magnesium, therefore releasing excessive amounts of Ca^2+^ in the environment and leading to the progression of the disease [40, 41, 43]. Thus, as magnesium seems to promote dissolution at high concentrations it supports the idea of self-regulation, when more Ca^2+^ is released, more calcium is bound to the biofilm strengthening its structure and less is left for remineralization [8]. Therefore, magnesium might not be the best agent to control the calcium flow in cariogenic biofilms.

Also zinc is a trace element with antibacterial properties and is present in the whole structure of the teeth [29], with the highest concentrations found in the outermost layer. Zinc is easily absorbed by the hydroxyapatite and can reach a position on the apatite crystal, making it resistant to acid dissolution. This could explain why the outer enamel layer is more resistant against caries than the subsurface [28] and why already a low concentration of Zn^2+^ inhibited bacterial adhesion (Fig. 3, Fig. 4), and had a strong antibacterial effect on mutans streptococcal growth (Fig. 1, Table 1) as well as on calcium dissolution (Fig. 2); leading to less biofilm and less EPS, ultimately resulting in a lesser cariogenic effect caused by mutans streptococci. Additionally, the titration data show that Zn^2+^ was having almost always the lowest affinity to all EPS used in this study (Table 2); thus, the unbound zinc could also still remain in the surrounding environment and keep its antimicrobial properties. Zinc binds the fructosyl site in the catalytic domain through an enzymatic action, leading to the inactivation of glucosyltransferases. Therefore, the glucan synthesis can be inhibited and thus, biofilm production reduced [30], which was also seen in Fig. 3 and Fig. 4. The effect of the reduced dissolution of hydroxyapatite (Fig. 2) has been shown earlier where in vitro studies have demonstrated the inhibitory effect of Zn^2+^ on the demineralization of hard tooth structure [44]. Of course, it must be mentioned that calcium found in the cariogenic cavity desorbs Zn^2+^ easily from hydroxyapatite and can therefore lead to alternate interactions that are not always easy to detect [29].

## Conclusions

The study illustrates how three divalent ions that are present in the upper layers of tooth surface are able to regulate the growth of mutans streptococci on the surface as well as when released in the surface or added externally to the surroundings affect the formation of cariogenic biofilms and their ability to dissolve tooth surface. There seems to be a fine balance between these ions that needs to be maintained as excessive concentrations of one or another destroy the healthy relationship between the three.

## Materials and methods

### Effect of divalent ions on the growth of mutans streptococci

Ten microliter stock solution of *S. mutans* (ATCC 25175) and *S. mutans* clinical isolate (CI [8];) were streaked on Columbia blood agar plates (BBL™, Becton Dickinson, Basel, Switzerland; supplemented with 50 mL/L of human blood, 0.5 mg/L of menadione, and 5 mg/L of hemin) and incubated aerobically at least 72 h at 37 °C.

Todd Hewitt medium was supplemented with either glucose (20 g/L; Sigma-Aldrich, Buchs, Switzerland), sucrose (20 g/L; Sigma-Aldrich, Buchs, Switzerland), or fructose (20 g/L; Sigma-Aldrich, Buchs, Switzerland) and colonies were resuspended to match McFarland 0.5 standard. Different concentrations (1, 3, 10, 30, 100 and 200 mM) of CaCl_2_, MgCl_2_, and ZnCl_2_ were added to the bacterial solution. A 48-well-plate was used to measure growth of bacteria in different solutions; for this 500 μL of prepared solutions was pipetted into the wells in duplicate and the plate was incubated at 37 °C for 48 h, aerobically in a plate reader (HTX Synergy, Biotek, Switzerland). Optical density (OD_600_nm) of the cultures was measured with intervals of 1 h, prior to each measurement the plate went through a short orbital shaking for 20 s with 500 rpm. All experiments were repeated three times in triplicates (*n* = 9).

The bacterial suspension with no divalent ions added served as a growth control and medium with no bacteria added was used as a blank measurement. All results were obtained as blank corrected growth curves over time. The data were analyzed using R-software and grofit package [45].

### Microscopic analysis of biofilm formations

The highest concentrations of divalent ions allowing bacterial growth were selected for biofilm formation on hydroxyapatite (HA) disks (5 mm in diameter; HiMed Inc., New York, USA). Bacterial suspension was prepared as described earlier and was supplemented with either 100 mM CaCl_2_ and 30 mM (glucose) or 10 mM MgCl2 (sucrose and fructose) and 1 mM ZnCl_2_ for *S. mutans* ATCC25175; and 10 mM CaCl_2_ and 10 mM MgCl_2_ and 1 mM ZnCl_2_ for *S. mutans* CI. Additionally, the samples used for confocal laser scanning microscopy (CLSM) later on were also incubated with 1 mM Alexa 647- dextrane-conjugate (Dextran, Alexa Fluor™ 647; 10,000 MW; Invitrogen™, Thermo Fisher, Reinach, Switzerland) in order to visualize the EPS formed in biofilms. Prior to biofilm formation, the disks were coated with 500 μL serum/saliva mixture (1:10) for 15 min at room temperature (RT) [34]. Thereafter, the disks were placed in bacterial suspension and incubated at 37 °C for 24 h. After that the samples were shortly dipped in 0.9% NaCl and prepared for CLSM.

The samples were placed in 500 μL 300 nM DAPI solution for 3 min to visualize the cells in biofilms, at RT in darkness. Thereafter, the samples were rinsed in 0.9% NaCl and placed upside down in Mowiol solution. All samples were imaged with Leica SP8 microscope (Leica SP8, Heerbrugg, Switzerland) using 63x (1.4x) oil immersion objective with Z-stack step of 0.3 μm. Three disks with biofilms were analyzed per condition (*n* = 3).

### Cariogenic dissolution of hydroxyapatite

Pikovskaya’s agar (5 g of HA powder, 0.5 g of (NH_4_)_2_SO_4_, 0.2 g of NaCl, 0.1 g of MgSO_4_ * 7 H_2_O, 0.2 g of KCl, 0.5 g of yeast extract, 0.002 g of MnSO_4_ * H_2_O, 0.002 g of FeSO_4_ * 7 H_2_O, and 15 g of agar, all dissolved it in 1 L of H_2_O) was used to measure the impact of different concentrations of (1, 10, and 100 mM) of CaCl_2_, MgCl_2_, and ZnCl_2_ of hydroxyapatite dissolution by *S. mutans* strains. Additionally, the medium was supplemented with either glucose (20 g/L; Sigma-Aldrich, Buchs, Switzerland), sucrose (20 g/L; Sigma-Aldrich, Buchs, Switzerland), or fructose (20 g/L; Sigma-Aldrich, Buchs, Switzerland).

A colony of *S. mutans* was inserted in the middle of the Pikovskaya’s agar and the plates were incubated aerobically for 10 days at 37 °C and thereafter, the dissolution zones were measured (*n* = 10).

### EPS extraction of S. mutans cultures

Ten microliter of the stock solutions (*S. mutans* ATCC 25175 and *S. mutans* clinical isolate), stored at − 80 °C, were inoculated into 5 mL Luria broth (BBL™, Becton Dickinson, Basel, Switzerland) and incubated aerobically for 20 ± 2 h, at 37 °C. Thereafter, 1 mL of the preculture of *S. mutans* clinical isolate and 4 mL of the preculture of *S. mutans* ATCC 25175 were inoculated into 1 L of 50% Luria broth supplemented with glucose, sucrose, or fructose (end concentration 20 g/L). Previously, the turbidity of the preculture, and thus the approximate number of bacteria, was measured with the Eppendorf BioSpectometer (OD_600_). The culture was incubated for 48 h under aerobic conditions 37 °C on a shaker set to 240 rpm.

The grown cultures were processed through glass-fiber filters (Millipore® Type 2 filters, retention 1,0 μm; Sigma-Aldrich, Buchs, Switzerland) and 0.22 μm filters (Millipore® StericupTM filter units PVDF membrane (Durapore); Sigma-Aldrich, Buchs, Switzerland). In order to obtain a precipitate of EPS, cold ethanol (Sigma-Aldrich, Buchs, Switzerland) had to be added in the ratio 2:1 to the solution. The precipitation was carried out for at least 12 h in a cold room at 4 °C.

The solution with the precipitated EPS was centrifuged the next day for 10 min at 3000 rpm (Sigma 4-16KS, Adolf Kühner AG, Birsfelden, Switzerland) at 4 °C. The liquid was discarded, and the recovered EPS was redissolved in ethanol and placed in dialysis bags (cellulose membrane, molecular weight cut-off 14′000 Da; Sigma-Aldrich, Buchs, Switzerland) to eliminate small molecular weight organic compounds. The dialysis was repeated two consecutive times against 3 l of 1 mM EDTA (Sigma-Aldrich, Buchs, Switzerland), followed by three dialysis against ultrapure water at 4 °C. Each of these steps lasted at least overnight. The dialyzed EPS was stored at 4 °C.

The concentration of EPS was measured by the phenol - sulphuric acid assay [46]. Briefly, 50 μL of the EPS was mixed with 500 μL deionized water, to this 25 μL of phenol was added and stirred properly. Thereafter, 2 mL of concentrated sulphuric acid was added as fast as possible. After 20 min the absorbance was measured at 490 nm (Eppendorf BioSpectrometer; Vaudaux-Eppendorf AG, Schönenbuch, Switzerland). In order to relate the values to concentrations, standard curve of 0.08% xanthan was also measured (0 μL, 20 μL, 40 μL, 60 μL 80 μL and 100 μL), *n* = 5.

### Analysis of the binding of the divalent ions to EPS

The binding of ZnCl_2_, MgCl_2_ and CaCl_2_ to EPS was analyzed by isothermal titration calorimetry (ITC). For every experiment the ITC ampoule was filled with 2.7 mL of EPS solution freshly dialyzed against the used buffer (see below) and the syringe with 300 μL divalent cation solution prepared in the same buffer. The ampoule with the EPS and the syringe with the divalent ions were placed in the TAM III ITC system (Waters / TA instruments, Delaware, USA), the titration parameters were defined as follows: 17 injections, each 15 μL over 15 s with an interval of 45 min. Blank titrations were made with the buffer used. Titration were performed using 30 mM Tris pH 8.5 as buffer for CaCl_2_ and MgCl_2_. For ZnCl_2_ deionized water was used due to precipitation of Zn oxydes or hydroxydes. Care was taken to adjust the pH of the water to the same value as EPS samples used. In addition to EPS samples, dextran was used as a commercially available polysaccharide as a reference compound. Binding parameters and affinity were calculated using the TA instrument embedded software (TAM assistant) and assuming a single equilibrium step. All experiments were repeated twice (*n* = 2).

### Statistical analysis

In order to identify differences between the control group not exposed to divalent ions and the other groups, data were analyzed by Student’s t-test with significance level set to *p* < 0.05 using GraphPad Prism 8.0. Normality test was performed in order to verify that the distributional assumptions were met for the t-test using the Shapiro-Wilk test for small sample size.

## Data Availability

The datasets used and/or analysed during the current study are available from the corresponding author on reasonable request.

## Abbreviations

DAPI: 4′,6-Diamidine-2′-phenylindole dihydrochloride
CI: Clinical isolate
CLSM: Confocal laser scanning microscopy
EPS: Exopolysaccharides
HA: Hydroxyapatite
ITC: Isothermal titration calorimetry
OD: Optical density
RT: Room temperature
